# 

**Published:** 1889-12-07

**Authors:** 


### SATURDAY, DECEMBER 7th, 1889.

**Secretaries and their Limitations** - 145
**Words of Consolation**—Fruit - 146
**The Proposed Registration of Nurses** - 147
**Among the Pines at Bournemouth** - 148
**Student's Column**—Victoria University - 148
**The Doctor's Pulpit.**—Physiological Candles - 149
**Darwinism on the Stage** - 150
**Hospital Abuse.** By HUGH WOODS, M.D. - 150
**Everybody's Page** - 151
**Notes and News** - 152
**Annotations.**—Martin Tupper—Beware of Chichester—Bad Smells - 154
**The Hospital Workshop.** XVII.—The Poplar Hospital 158
**Fallow Crops.**—Charity's Financial Outlook - 159
**The Practitioner's Outlook and Retrospect:**
MEDICINE—Communicability of Cerebrospinal Meningitis—Absinthe—Tubercle Infection—Muscular Atrophy in Tabes Dorsalis - 155
Iodides for Scrofulous Children—Treatment of Anæmia by Enemata of Defibrinated Blood—Renal Lesions - 156
SURGERY—Tonsil Slitting - 156
THERAPEUTICS—No More Insomnia - 156
Accumulation in the Organism of Bromide of Potassium—Hypnotism - 157
WITHIN THE WARDS—Uterine Cancer - 157
NEW DRUGS, APPLIANCES, AND THINGS MEDICAL—Dahl's Pure Milk - 158
**Scraps and Gleanings** - 160

### EXTRA SUPPLEMENT—THE NURSING MIRROR.

**En Passant.**—Incorporation of the Midwive's Institute—Marriage and the Pension Fund—Asylum Attendants—Scandals—Something New in Journalism—Where THE HOSPITAL can be Bought—American Notes - xli
**The Nurses' Bookshelf.**—Masso-Therapeutics—Our Toiling Sisters - xliii
**The Show of Nurses' Uniforms** - xlii
**Everybody's Opinion.**—A First Experience, xliii; Co-operative Registry for Nurses - xliv
**Fair Dispensers—Notices—Notes and Queries** - xliv
**Presentations—Vacancies** - xliv
**Amusements and Relaxation** - xliv

